# The “Federica” Hand

**DOI:** 10.3390/bioengineering8090128

**Published:** 2021-09-21

**Authors:** Daniele Esposito, Sergio Savino, Emilio Andreozzi, Chiara Cosenza, Vincenzo Niola, Paolo Bifulco

**Affiliations:** 1 Department of Electrical Engineering and Information Technologies, Polytechnic and Basic Sciences School, University of Naples “Federico II”, 80125 Naples, Italy; emilio.andreozzi@unina.it (E.A.); paolo.bifulco@unina.it (P.B.); 2 Department of Neurorehabilitation, IRCCS Istituti Clinici Scientifici Maugeri, 27100 Pavia, Italy; 3 Department of Industrial Engineering, Polytechnic and Basic Sciences School, University of Naples “Federico II”, 80125 Naples, Italy; sergio.savino@unina.it (S.S.); chiara.cosenza@unina.it (C.C.); vincenzo.niola@unina.it (V.N.)

**Keywords:** active hand prosthesis, 3D-printing, differential force distribution, force-myography control, vibrotactile sensory feedback

## Abstract

Hand prostheses partially restore hand appearance and functionalities. In particular, 3D printers have provided great opportunities by simplifying the manufacturing process and reducing costs. The “Federica” hand is 3D-printed and equipped with a single servomotor, which synergically actuates its five fingers by inextensible tendons; no springs are used for hand opening. A differential mechanical system simultaneously distributes the motor force on each finger in predefined portions. The proportional control of hand closure/opening is achieved by monitoring muscle contraction by means of a thin force sensor, as an alternative to EMG. The electrical current of the servomotor is monitored to provide sensory feedback of the grip force, through a small vibration motor. A simple Arduino board was adopted as the processing unit. A closed-chain, differential mechanism guarantees efficient transfer of mechanical energy and a secure grasp of any object, regardless of its shape and deformability. The force sensor offers some advantages over the EMG: it does not require any electrical contact or signal processing to monitor muscle contraction intensity. The activation speed (about half a second) is high enough to allow the user to grab objects on the fly. The cost of the device is less then 100 USD. The “Federica” hand has proved to be a lightweight, low-cost and extremely efficient prosthesis. It is now available as an open-source project (CAD files and software can be downloaded from a public repository), thus allowing everyone to use the “Federica” hand and customize or improve it.

## 1. Introduction

The hand is the principal effector organ of the upper limb, thanks to which humans can perform many functions in daily life. Hand loss is a traumatic event, which involves the loss of motor and sensory functions, with inevitable disability. Hand prostheses are intended to restore both the appearance and some functionalities of the hand in people with amputations or congenital malformations. Especially in poor and developing countries, there may be many additional causes of amputation, associated with trauma from war, explosives, or industrial or environmental accidents, the consequences of which are often aggravated by a lack of public health care [1,2,3]. All over the world, many people cannot afford high-tech, commercial hand prostheses, so various low cost solutions have been proposed [4,5,6]. The local availability of materials, ease of realization, effectiveness, robustness, durability, and free access to the hardware and software should be taken into account in the design process. The “Federica” prosthetic hand was specifically designed to meet these needs [7,8,9,10,11]. “Federica” restores the grasping functions that are the most useful in everyday life. Its design is focused on achieving an energy-efficient operation, as well as providing a fast and responsive control system with sensory feedback capability that allows the user to rapidly become confident controlling the prosthesis. An ad hoc-designed mechanical system, together with a novel technique for monitoring muscle contraction, provide a very fast power grasp, also simplifying the learning curve for the user. During the design process, the low cost of realization has been pursued by conceiving almost all components to be 3D-printable (only one off-the-shelf servomotor must be purchased); also, modularity has been promoted in the design to simplify its assembly, maintenance and repair operations.

The control of active prostheses is usually obtained by monitoring residual muscle activity, and electromyography (EMG) is by far the most-used technique [12,13]. EMG signals are generally acquired by means of dry electrodes embedded in the prosthesis socket. State-of-the-art myoelectric-controlled prostheses generally use two EMG channels [13,14]. The raw EMG signal has to be processed to obtain a suitable signal for prosthesis control: to this aim, the EMG linear envelope (EMG-LE) is usually computed, since it provides concise information on developed muscle strength. Previous studies showed that a force-sensitive resistor (FSR), positioned via a mechanical coupler on the skin above a specific muscle, provides a signal (referred to as forcemyogram, FMG) that is extremely similar to the EMG-LE signal obtained from the same muscle [13,15,16,17]. Such high similarity suggested the potential use of FMG as an alternative to EMG for prosthesis control. FMG offers some advantages over EMG: it does not need electrodes and stable electrical contact; it has much lower susceptibility to electromagnetic interferences; it does not need signal processing to obtain a reliable control signal (the FMG signal can be used as-is); it has a simpler conditioning circuit; the extreme compactness and thinness of its sensor, which improves wearing comfort; and its lower cost. Furthermore, the simplicity of FMG signals made possible the use of low-cost and open-source Arduino boards as prosthesis controllers.

“Federica” is powered by a single servomotor and performs the power grasp by means of a custom underactuated differential mechanical system. Many underactuated mechanical systems (less actuators than degrees of freedom [DOF]) have been proposed to simplify the control of hand prostheses, without abandoning its mimicry of the dexterity of some natural hand movements. Underactuated mechanisms aim to reduce the number of active DOF and to reproduce common hand actions without increasing mechanical and control complexity [18]. Moreover, underactuated mechanisms do not control a single element (e.g., a phalanx), but rather a set of elements (e.g., a finger or some fingers).

Examples of underactuated systems include floating pulley trees and differential gearings. These systems turned out to be very useful in grasping tasks, where the fingers should be able to conform with irregularly shaped objects [19]. Different studies [18,19,20] presented prosthetic devices with multiple DOF, controlled with a limited number of actuators and a differential mechanism. The resulting underactuated mechanism allows to transfer the motor force from few actuators to many phalanxes, taking advantage of self-adaptive grip capacity and a lower manufacturing cost [20]. Adaptive grasp is the ability of the fingers and thumb to adapt to the shape of an object, in order to increase the number of contact points between the hand and the object [18]. A reduced number of actuators also implies simplification of the prosthetic control system and can be advantageous for the patient. The user would prefer to send few, fast and direct commands to the prosthesis in performing normal daily activities, without the need to commit to much in the control [18].

A specific underactuated mechanism [20] was presented to control five fingers (15 DOF) by means of one actuator, with the aim to grab complexly shaped objects and to allow appropriate force distribution. Each finger has three compliant joints, with rotation and spring functions that are not suitable for grabbing heavy objects because they can easily be abducted or adducted [20]. Another prosthesis, named TUAT/Karlsruhe humanoid hand, adopted a mechanism for the cooperative movement of finger and palm joints [21]. To achieve functionality allowing the operation of each finger individually, without losing the conventional grasping function, the main servomotor cooperates with six small servomotors, arranged between the metatarsals. By using springs or rubber bands, each finger can return to its rest position [22]. A three-fingered prosthesis, called the SPRING hand [18], was shown to be capable of self-adaptive grasping by means of an underactuated mechanism. The eight DOF of the hand are driven by one motor, and each finger includes cables and springs, in order to guarantee the shape adaptation to the grasped object [18]. A project entitled KIT Prosthetic Hand [23] presented an underactuated system in which its four fingers are simultaneously driven via a force-distributing transmission based on the TUAT/Karlsruhe mechanism [21,22], while the thumb is actuated by a second motor. Custom made springs in every joint ensure the passive reopening of the fingers [23]. A mechatronic hand, with five fingers and controlled by a single actuator, was shown to be able to perform four types of grasps, and to switch between them by means of a bistable ratchet coupled to the thumb adduction/abduction [19].

All the aforementioned [19,20,22,23] devices made use of underactuated mechanical systems combined with springs and/or elastic elements, which unavoidably absorb a considerable amount of energy supplied by the motors when the hand closes. In general, elastic elements are used for the passive opening of the hand (i.e., fingers extension), while, during the closing of the hand (i.e., fingers flexion), springs have to be loaded, and this requires energy expenditure [24].

However, multiple-DOF devices with multiple actuators are also presented in other studies. An example is the fully actuated ROBIOSS hand [25], consisting of four fingers, each with four DOF, driven by 16 actuators; another example is the doubly actuated Utah–MIT hand [26], which includes four fingers, each with four DOF and a three-DOF wrist, driven by 38 separate actuators. Obviously, the complexity of both the mechanics and the control are considerably increased with respect to the underactuated devices.

A further issue addressed in the “Federica” hand project was the development of a system to provide a non-invasive sensory feedback of grip force. Amputation involves the loss of sensory receptors and the interruption of the physiological channels, through which stimuli are normally perceived and transmitted to the central nervous system (CNS) [27,28]. Restoration of the sensory function is an important challenge faced by prostheses designers. There are two possible ways to elicit sensory feedback [27,29,30]: invasively, by using neural electrodes implanted in the peripheral nervous system, in afferents originally serving the fingers and palm, in order to directly interface with the CNS; or non-invasively, by providing feedback to residual sensory systems (e.g., electrotactile and vibrotactile stimulations on the residual limb, etc.). In both cases, the user should be trained to associate stimuli with physical events occurring at the prosthesis (exteroception) or to states of the prosthesis (proprioception) [27,29]. A prosthesis with sensory feedback uses a closed-loop control, providing the user with both exteroceptive and proprioceptive information. However, commercial hand prostheses used in clinical practice are generally not equipped with such closed-loop control and, consequently, the user needs to rely on visual feedback and incidental stimulation (hearing, socket pressure, etc.) [27]. An exception is the VINCENTevolution2 prosthetic hand, which is equipped with haptic feedback [28,31]. Other devices like Sensor-Hand, i-Limb and BeBionic use sensors to measure and automatically regulate the grip force, without including the user in the loop; in particular, i-Limb and BeBionic exploit motor current sensors with the aim of achieving an adaptive grasp [27]. Nevertheless, experimental projects have mainly proposed electrotactile and vibrotactile stimulations to code information relative to grasp and provide the user with non-invasive sensory feedback [27]. Electrotactile stimulation evokes sensations by passing a local electric current to stimulate afferent nerves in the skin with surface electrodes. The modulated parameters include frequency, amplitude and pulse width [30]. Electrocutaneous feedback can cause pain for the user, and can also create interferences in prostheses controlled by EMG signals [29]. Vibrotactile stimulation is achieved by providing the user with mechanical vibration on their skin to convey tactile sensations [30]. The two main parameters of the stimulus are vibration amplitude and frequency [27]. The use of vibrotactile feedback was reported to improve user performance via better grip-force control and fewer errors when performing daily activities [27].

A different approach to providing sensory feedback is based on mechanotactile stimulation, consisting in a force/pressure applied in a different area (the residual limb) from the original stimulus [30]. An example is an elastic armband connected to two DC motors [32], which rotate in opposite directions to tighten or loosen the band, in order to help the user adjust the gripping force. Current mechanotactile devices are more cumbersome and heavier than vibrotactile or electrotactile devices, also resulting in higher energy consumption [30].

However, sensory feedback could encourage a sense of body ownership for prosthetic users and help them to correctly apply force in their grip; therefore, restoring a kind of tactile sensation is helpful in improving user experience as well as handling performance [30].

This study presents the “Federica” hand, an anthropomorphic and adaptive prosthetic device that minimizes the complexity of all components without sacrificing much of the performances. Despite being powered by a single, off-the-shelf servomotor, it has shown high versatility in gripping disparate objects (even deformable ones), very high energy efficiency (for an anthropomorphic prosthesis) [24] and a speed not matched by any other prosthetic prototypes [11]. The control system, based on customized FSRs sensors, is innovative, more user-friendly and faster than EMG-based control [14]. Obviously, the proposed solution does not intend to compete with sophisticated research or commercial products. Rather, it is an inexpensive prosthesis, for people in need, featuring various advantageous solutions that could be used by the scientific community in other projects as well. For this purpose, the list of components, CAD files for 3D printing, all the electronics and the sensor design are provided as Supplementary Materials https://www.doi.org/10.6084/m9.figshare.13480248 (accessed on 16 September 2021). Further information and video about the “Federica” hand project are available at: http://ingegneria-biomedica.dieti.unina.it/index.php/en/projects/federica-prosthetic-hand.html (accessed on 16 September 2021).

## 2. Materials and Methods

### 2.1. Mechanical Design Specifications

**Figure 1:**
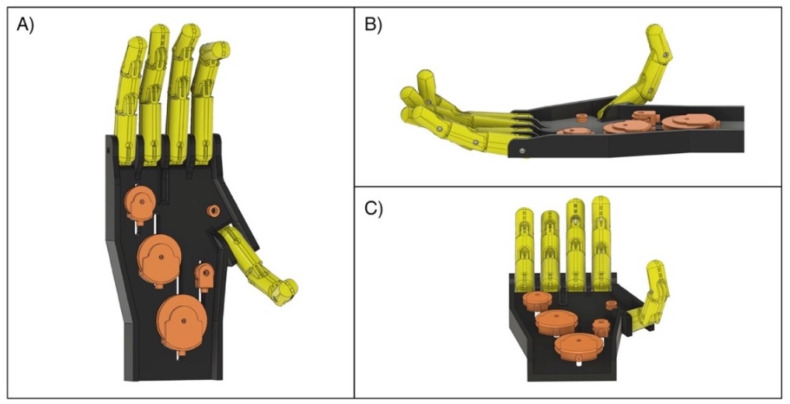
3D renderings of the “Federica” prosthetic hand: (**A**) Top view; (**B**) Side view; (**C**) Bottom view.

The parts of the “Federica” hand are almost completely 3D printed in polylactic acid (PLA) (project files are available as Supplementary Materials). The prosthesis has an anthropomorphic appearance and is composed of five fingers, each with three phalanges. It is strongly underactuated, as a single servomotor (180 degrees metal steering gear, 30 kg/cm torque) serves to synergistically move the fifteen phalanges (15 degrees of freedom) by means of inelastic cables (artificial tendons). A custom differential mechanical system allows a balanced and constant distribution of the force between the fingers (see Figure 1).

**Figure 2:**
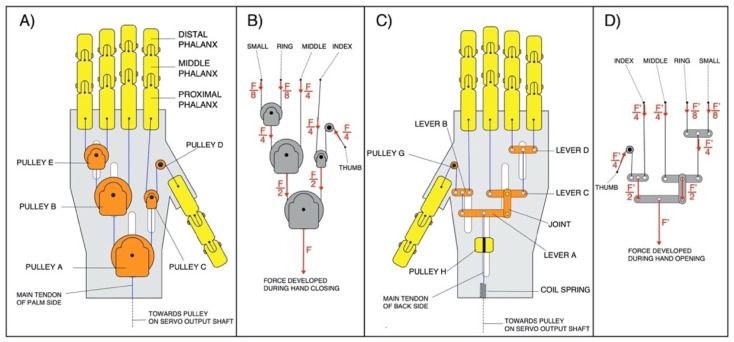
Illustration of the “Federica” hand design: (**A**) Palm side of the mechanical system; (**B**) force distribution on its palm side during closing; (**C**) the back side of the mechanical system; (**D**) force distribution on back side during opening.

On the hand’s palm side (see Figure 2A,B) it is possible to observe that the servomotor exerts a force $\vec{F}$ on the pulley A; here this force is divided into equal force components ($\vec{F}/2$) towards the pulleys B and C; pulley B further divides the force at its input into two equal force components, ($\vec{F}/4$), directed towards the middle finger and pulley E, respectively; pulley E, in turn, divides the force at its input and supplies the same force components, ($\vec{F}/8$), to the ring and little fingers; finally, the pulley C distributes the force at its input into equal force components, ($\vec{F}/4$), directed towards the index finger and thumb. This particular design allows to always have $\vec{F}/4$ on the thumb, index and middle fingers, and $\vec{F}/8$ on both the ring and little finger. A former functional–anatomical analysis of the human hand [33] revealed that a greater reliability in the grip is given by the thumb (for its opposability), index and middle fingers; while the ring and little fingers assist with prehension.

**Figure 3:**
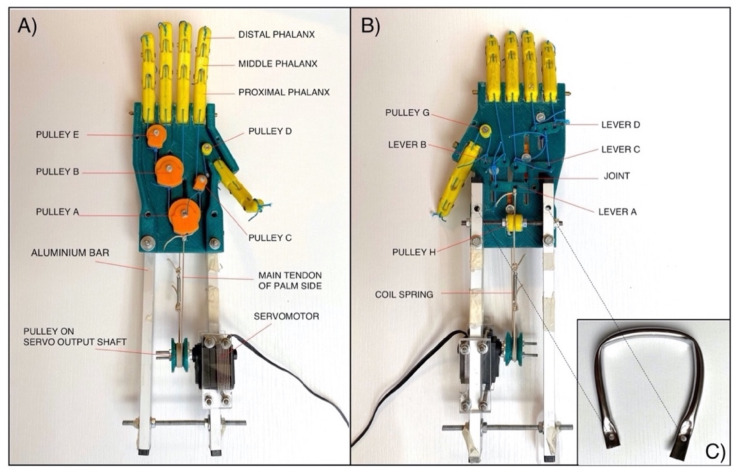
Current version of the “Federica” hand: (**A**) Palm view; (**B**) Back view; (**C**) Handle. Two aluminum bars were mounted on the back side of the prosthesis to remotely fix the servomotor.

The mechanical system is equipped with two main tendons: one actuator (palm side) and one antagonist (back side) to perform, respectively, the closing (fingers flexion) and opening (fingers extension) of the hand. Both tendons are connected to the same pulley on the servomotor’s output shaft (see Figure 3A), so that, when one is pulled, the other is released, and vice-versa. In particular, in the back side (see Figure 2C,D), the traction force used for the extension of the fingers is distributed by using levers. A coil spring with low elastic constant (K ≈ 2.5 N/mm) is inserted on the main tendon of the back side (see Figure 2C), in order to allow the complete flexion of the fingers, which is otherwise not possible by using inextensible cables (tendons). 

It is interesting to emphasize that most of the hand prostheses on the market [34,35] use different coil springs positioned inside the fingers to permit their passive extension. This entails a considerable energy absorption in having to overcome the elastic forces exerted by these coil springs during hand-closing tasks.

On the contrary, the “Federica” hand actively performs its hand-opening and closing movements in a closed chain system: the traction of the main tendon from the palm side allows the hand’s closure, while the traction of the main tendon from the back side permits its opening. A powerful grip is made possible by particularly efficient energy transfer from the motor to the prosthetic fingers [24].

The current version of the “Federica” hand is shown in Figure 3. In order to preliminarily test it on healthy subjects, a rigid handle was mounted on the back of the prosthesis (see Figure 3B,C), while two aluminum bars were used to remotely fix the servomotor to it. It is clearly observable that the main tendon from the palm side, after some winding around the pulley on the servomotor output shaft, is joined with the main tendon of the back side, thus making the closed chain of the mechanical system.

**Figure 4:**
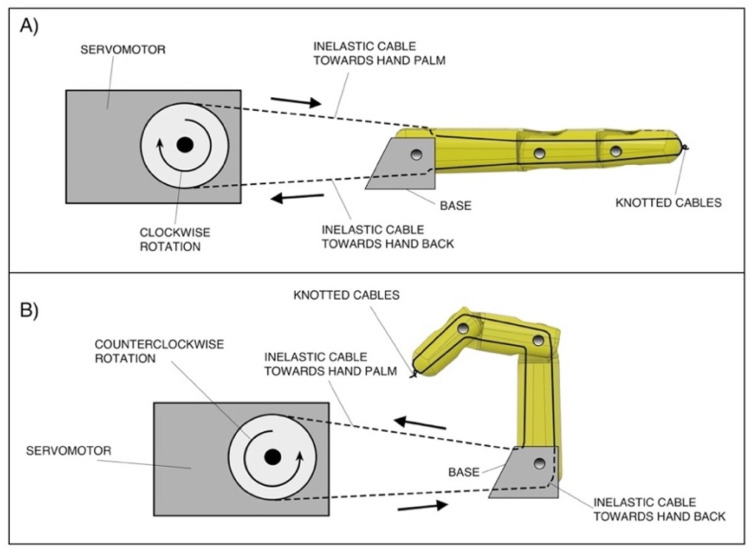
Finger-actuation mechanism: (**A**) finger extension by traction on the inelastic cable toward the hand back; (**B**) finger flexion by traction on the inelastic cable toward the hand palm.

As a further example of the closed chain of this mechanical system, Figure 4 shows the actuation mechanism of a single prosthetic finger. The clockwise rotation of the servomotor, by traction on the inelastic cable towards the hand’s back (Figure 4A), performs finger extension; on the other hand, the counter-clockwise rotation of the servomotor, by traction on the inelastic cable towards the hand’s palm (Figure 4B), performs finger flexion. The figure also highlights that these inelastic cables pass inside each phalanx (through special holes) and are knotted at the fingertip.

The design specifications of the “Federica” hand are: that the size should be suitable for an adult man, yet scalable; that the prosthesis should be as light as possible; that it should be able to effectively help in the actions of daily living (ADL); that the prosthesis should be as energy-efficient as possible, with the least complexity and lowest cost possible; and that a simple sensing function should be optionally added.

### 2.2. Force-Myographic Control and Vibrotactile Feedback System

**Figure 5:**
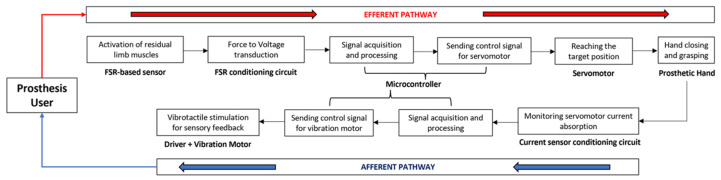
Flowchart describing the efferent pathway (force-myographic control system) and the afferent pathway (vibrotactile sensory feedback system).

Figure 5 shows the closed loop for the prosthesis user, made by combining the force-myographic control and the vibrotactile sensory feedback. In particular, both the “efferent pathway” (from the contraction of the target muscle to the execution of the grasp action) and the “afferent pathway” (from the sensing of the servomotor current absorption to the vibrotactile stimulation for the sensory feedback of the grip force) are presented.

**Figure 6:**
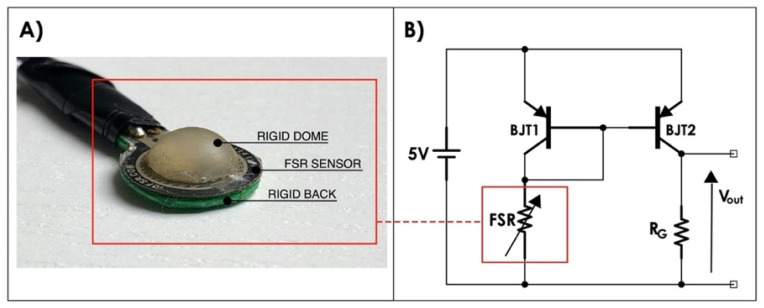
(**A**) A picture of an FSR-based sensor equipped with a custom mechanical coupler; (**B**) FSR conditioning circuit, based on current mirror (R_G_ = 910 Ω).

The force-myographic control signal for prosthesis activation, is provided by a custom piezoresistive force sensor based on FSR 402 [10,11,14,16] (see Figure 6A), positioned in correspondence with the forearm muscles assigned to the control. The positioning of the sensor depends on both the shape of the forearm stump and the location of the residual muscles. The FSR-based sensor is extremely thin: even with the mechanical coupler (the rigid dome in Figure 6A), its thickness is limited and this allows for its positioning both inside the socket or on other control muscles.

The FSR-based sensor is conditioned by means of a current mirror circuit [10], itself realized by means of a pair of pnp BJTs (2N2907), glued to the two-transistor cases, in order to maintain them at approximately the same temperature. The circuit replicates the FSR current in the gain resistor R_G_ (see Figure 6B) [10] providing an output voltage that is directly proportional to the force exerted on the sensor.

A static calibration of the FSR-based sensor was carried out to evaluate the relationship between the muscle force exerted on the sensor and the voltage output from the conditioning circuit (Figure 6B). Different calibrated weights were progressively applied on the active area of the sensor and the corresponding output voltages were recorded; the static calibration relationship was then obtained by means of linear regression.

**Figure 7:**
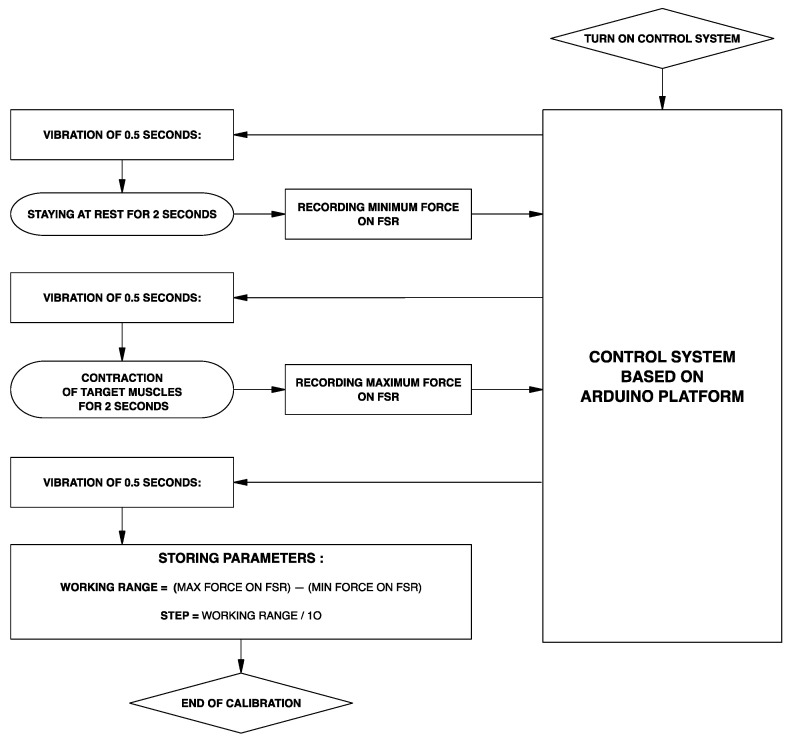
Flowchart describing the calibration phase of the control system.

The control system was implemented on an open-source Arduino platform (Arduino Nano). The whole system is powered by a 7.4 V battery pack. Each time the prosthesis is worn, a simple calibration procedure is recommended: while remaining at rest for two seconds, the mean minimum force value applied on the FSR-based sensor is acquired and stored; then, while exerting the maximum voluntary contraction for two seconds, the mean maximum force is acquired and stored. The user can be guided in this calibration phase by means of a vibration motor (or by a buzzer), which signals when the patient must contract the muscle. Figure 7 depicts the entire calibration phase as a flowchart. After calibration, the prosthesis is ready to work.

Proportional control is achieved by associating different levels of muscle contraction (from the minimum to the maximum of the FSR’s output voltages) to the rotation angle of the servomotor (from hand open, at 0 degrees, to hand closed, at 180 degrees). Even few levels (e.g., ten) allow a progressive and smooth hand closure.

The “Federica” hand is equipped with an optional sensory system that provides information to the user about the exerted grip force. An estimate of the grip force is obtained by measuring the electric current absorbed by the servomotor, then this information is provided to the user through a vibration transducer. A previous study [9] has already shown that the mechanical torque generated by the servomotor is highly correlated to its current absorption. Currently, the servomotor’s characterization is carried out by applying, in suspension, different calibrated weights to its arm and measuring the related absorbed currents. Torque [Ncm] is computed by multiplying the weight (equivalent to the force [N]) by the length of the servomotor arm, since the arm and the applied force form a 90 degrees angle.

**Figure 8:**
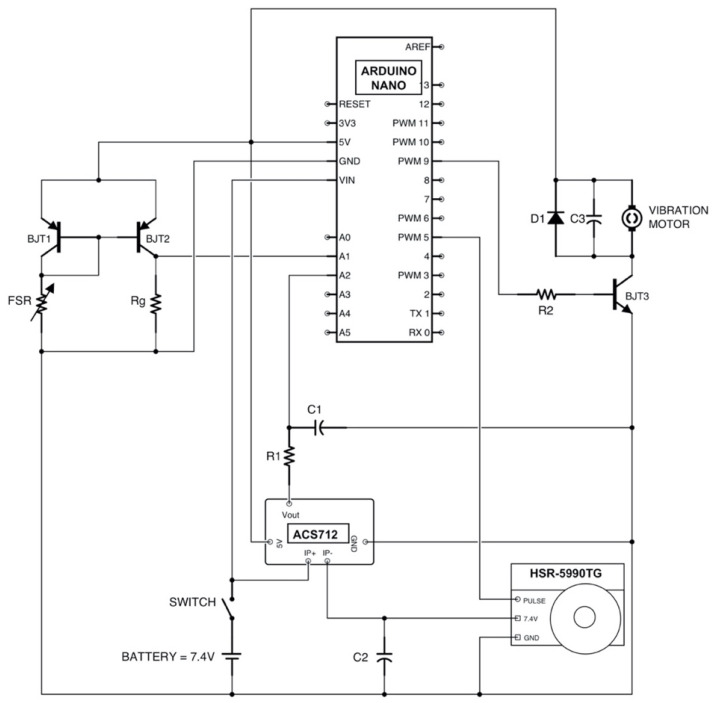
Circuit diagram based on Arduino platform (R_G_ = 910 Ω; R_1_ = 1.6 KΩ; C_1_= 20 μF; C_2_ = 0.47 mF, D_1_ (1N4001); R_2_ = 1KΩ; C_3_ = 0.1 μF).

The motor current is continuously monitored through the ACS712 Hall effect sensor [11]. When the absorbed current exceeds a certain threshold for a given time (double threshold criterion [36]), a vibration is provided to the user by means of an eccentric motor placed in contact with the patient’s skin (on–off sensing). In particular, an increase in current of 200 mA compared to the steady motor absorption, for a period greater than 200 ms, was empirically chosen to trigger the vibration motor. Likewise, the vibration ends when the current absorption falls beneath the same threshold for more than 200 ms. In a previous study [11] it has already been shown that the servomotor, according to its structure and controller, absorbs current in a non-continuous way and, consequently, the absorbed current continuously shows impulsive variations. The ACS712 output signal is low-pass filtered at 5Hz by means of a hardware RC filter (R_1_C_1_ in Figure 8), in order to give the Arduino board a more stable signal.

The simple circuit to drive the vibration motor is showed in a section of Figure 8. A npn BJT (BJT3) is used to amplify the driver current supplied from the Arduino to the vibration motor; on the other hand, a 1 KΩ resistor (R_2_) in series with the base of the transistor, is also used for limiting this current and avoiding motor damages. The diode (D_1_) acts as a surge protector for the microcontroller against voltage spikes that the motor could produce while rotating, and a 0.1 μF ceramic capacitor (C_3_) absorbs these voltage peaks.

**Figure 9:**
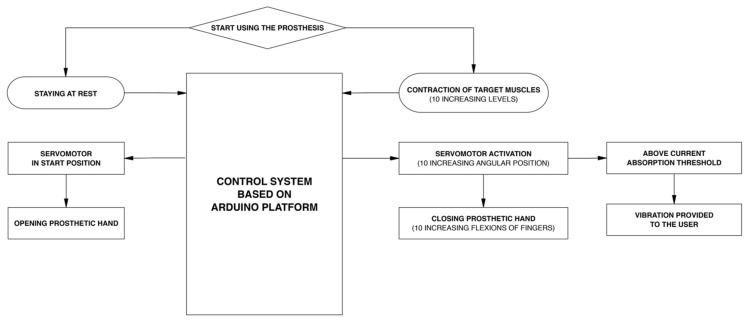
Flowchart describing the logic of the software implemented on the Arduino board.

The flowchart of Figure 9 describes the software operations implemented on the Arduino board, for controlling the servomotor and the vibration motor (see the Supplementary Materials for source code).

## 3. Results

### 3.1. Current Realization of “Federica” Hand

The design specifications for the “Federica” hand are outlined in Table 1. The size was specified to be suitable for an adult male and the mass of the device (only the prosthetic hand) is about 200 g. Different tasks of daily life, carried out in a previous study [11], showed the capability of the device to grasp differently shaped objects, raising loads of up to 1 Kg, and an activation speed of about 0.5 s, from the muscle sensor trigger to the complete closure of the hand. Moreover, using batteries of 7.4 V with a capacity of 3000 mAh, and considering various daily activities performed by the patient, it was estimated that the prosthesis would have an autonomy of at least an entire day [11]. A further previous study [24] showed that the mean grip force of the “Federica” hand is 8.80 N; while, on average, the middle phalanges exert a force of 2.65 N and the distal phalanges a force of 1.66 N. Moreover, the force-transfer ratio (grip force/actuator tendon force) of the whole mechanical system is about 12.85%, and the mean dissipated energy for a complete cycle of closing–opening is 106.80 Nmm, a result lower than that of many commercial body-powered prostheses.

The approximate costs (overestimated) for the realization of the prosthetic device (a total of 100 USD) are shown in Table 2. The most expensive component is the Hitec HSR-5990TG servomotor, because it is equipped with metal gears and allows the generatation of remarkable mechanical torque (30 Kg/cm). Conversely, the costs related to the 3D-printed components and the realization of the entire control system are much more affordable.

### 3.2. Static Calibration of the FSR-Based Sensor

**Figure 10:**
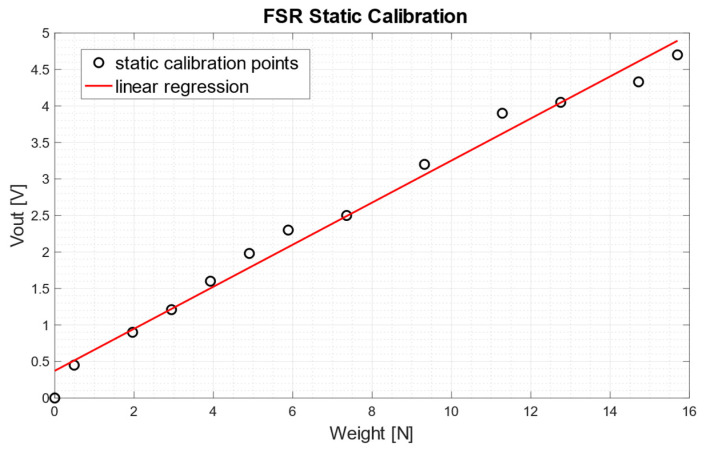
FSR-based sensor static calibration: scatter plot of the experimental data (o) and regression line.

Results of the static FSR calibration are showed in Figure 10. Experimental measurements are represented as circles, while linear regression is represented as a red continuous line. Considering the weights applied to the sensor to be *x* (N) and the output voltage from the conditioning circuit to be *y* (V), the equation of the linear regression resulted:

(1) y=0.288 x+0.3727

with a coefficient of determination R^2^ equal to 0.984.

### 3.3. Servomotor Characterization

**Figure 11:**
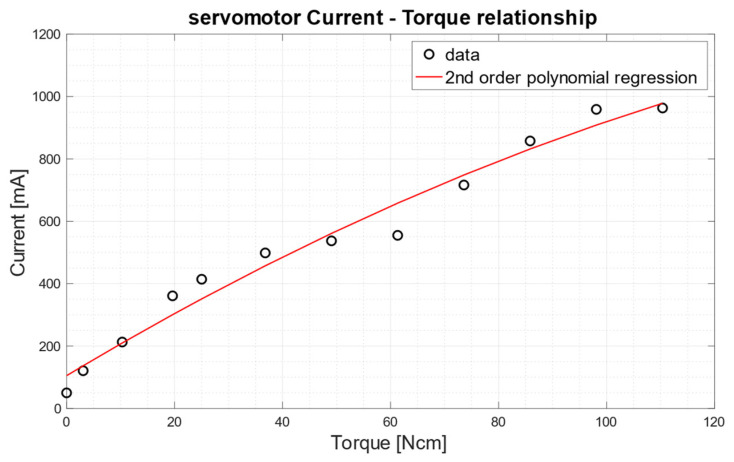
Relationship between the current absorbed by the servomotor (measured in mA) and the torque generated by it (measured in N·cm). The second-order polynomial regression function is depicted as a red continuous line.

Figure 11 shows the trend of the current absorbed by the servomotor Hitec HSR-5990TG as a function of the mechanical torque.

The relationship was approximately linear to about 100 N·cm, whereas, for higher torques, the absorbed current saturates at about 950 mA. A second-order polynomial function well fits the current–torque scatter plot, with a coefficient of determination R^2^ equal to 0.973. The equation of this second-order polynomial regression function is:

(2) $$\mathrm{Current}\;\left[\mathrm{mA}\right]=104.9+10.41\times \mathrm{Torque}\;-0.023\times {\mathrm{Torque}}^{2}$$

### 3.4. FMG–EMG Comparison

**Figure 12:**
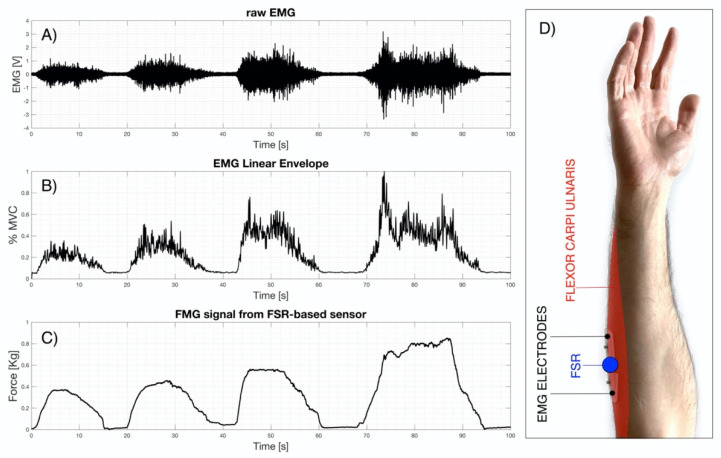
Simultaneous recordings from the forearm muscle “flexor carpi ulnaris”, while performing four wrist flexion movements with increasing strength: (**A**) Raw EMG signal; (**B**) EMG linear envelope; (**C**) FMG signal from FSR–based sensor; (**D**) FSR–based sensor and EMG electrodes positioned on the flexor carpi ulnaris of the subject’s forearm.

EMG and FMG signals were acquired simultaneously while performing wrist flexion movements (10 kHz sampling frequency with 16-bit precision, by means of the National Instruments PCI-6251). EMG electrodes were positioned on the forearm muscle “flexor carpi ulnaris”(according to SENIAM guidelines [37]), while the FSR-based sensor was placed between the EMG electrodes (see Figure 12D). The FSR was locally fixed on patient’ skin by means of elastic adhesive tape. The voluntary subject performed four wrist flexion movements with increasing strength (until to maximum voluntary contraction [MVC]), holding the position for several seconds and then returning to a relaxed state.

In detail, Figure 12 shows: in panel (a) the raw EMG signal, acquired by means of a biopotential amplifier (Biomedica Mangoni BM623) enabling a hardware 20–500 Hz band-pass filter and a gain of 1000 V/V; in panel (b) the EMG linear envelope, obtained by applying full-wave rectification followed by a low-pass filter on the raw EMG signal (Butterworth 4-rd order, 5 Hz cut-off frequency); in panel (c) the FMG signal obtained as raw output from the conditioning circuit of the FSR-based sensor (see Figure 6B); finally, in panel (d), the subject’s forearm with the EMG electrodes and the FSR-based sensor on the monitored muscle.

To quantitatively measure the relationship between the EMG linear envelope and the FMG signal, Pearson’s correlation coefficient “r” was computed and scored 0.9 (*p*-value < 0.0001 (two-tailed test)).

However, for further details about FMG–EMG comparison, we suggest referring to previous studies [14,16].

### 3.5. Grip Force Sensing

**Figure 13:**
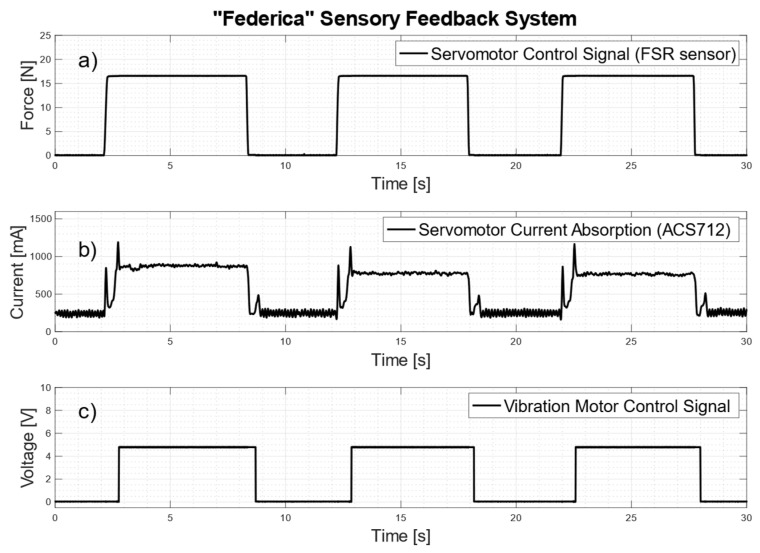
Simultaneous recordings of: (**a**) prosthesis control signal (output of the FRS-based sensor conditioning circuit); (**b**) current absorbed by the servomotor (output of the ACS712 conditioning circuit); (**c**) vibration motor control signal.

Figure 13 shows simultaneous recordings of FSR-based sensor output (N), servomotor current absorption (mA) and vibration motor control signal (V). The FSR-based sensor (see Figure 13a) was triggered by exerting a first impulsive and then constant force (about 16N), in order to quickly activate the closure of the prosthetic hand and keep the grip on an object for some seconds. The current absorbed (see Figure 13b) by the servomotor shows a first peak at the motor starting (to overcome static friction) and a second peak when the prosthetic fingers reach the object; then, the current value stabilizes at about 800 mA for the entire holding time. The panel (c) of Figure 13 shows the activations of the vibration motor, corresponding to three consecutive grasps.

### 3.6. Test on Healthy Subjects

**Figure 14:**
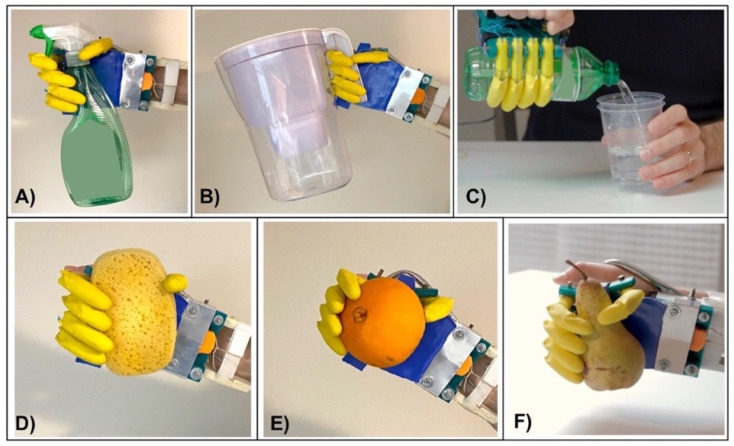
“Federica” hand tested on healthy subjects, while holding: (**A**) a spray cleaner; (**B**) a water tank; (**C**) a bottle; (**D**) a sponge; (**E**) an orange; (**F**) a pear.

Various tests were carried out to verify the efficacy of the “Federica” hand in performing some actions of daily living (ADL), some of which have been reported in previous studies [9,10,11,16,24]. Healthy volunteers wore the “Federica” hand, holding the rigid metallic handle, suitably mounted on the back of the prosthesis, and some bands were secured to their forearms by the two aluminum bars (see Figure 3). The FSR-based sensor (see Figure 6) was applied on the flexor carpi ulnaris of each subject, by means of an armband. The subjects performed daily actions as to grab objects (both rigid and deformable) of different shapes, as showed in Figure 14. These tests proved the self-adapting capacity of the prosthetic device to grab many complex shaped objects (both rigid and compliant), ensuring a secure grip on them.

### 3.7. Comparison between “Federica” Hand and Other Hand Prostheses

The following section reports characteristics, functionalities and performances of the “Federica” hand compared with other prosthetic hands. In detail, Table 3 shows a comparison between “Federica” and other externally powered hand prostheses: both experimental prototypes (developed by universities or research institutes) and commercial devices [34], in terms of weight, grasp speed, grip force, DOF, number of actuators, kind of grasps (power, intermediate, precision [38]), adaptive grip, price, etc.

Furthermore, Table 4 displays the comparison between “Federica” and body powered hand/hook prostheses (already presented in a previous study focused on “Federica” hand performances [24]), regarding the works for hand opening, closing and hysteresis (energy efficiency).

## 4. Discussion

The simple, 3D printed, low-cost, myo-controlled “Federica” hand, especially intended for research studies and use in developing countries, was presented in this paper. This study provides the readers with all the knowledge necessary for the reproduction of the device; therefore, the CAD modelling files of the 3D printable components and the software developed for the Arduino platform are supplied as Supplementary Materials. The hardware and software are proposed to be public and free.

**Figure 15:**
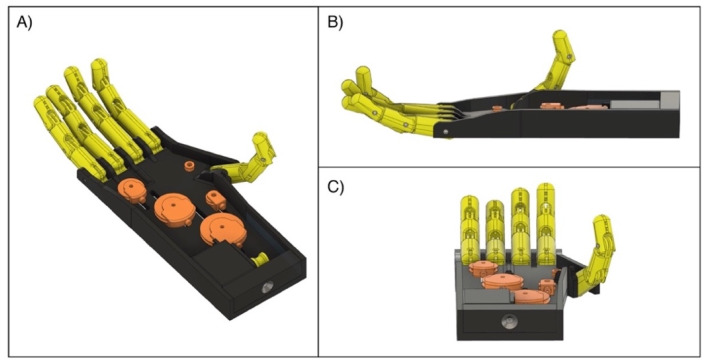
3D renderings of the “Federica” hand with built-in servomotor: (**A**) top view; (**B**) side view; (**C**) bottom view.

The current dimensions of the device are suitable for an adult male, but the dimensions can be easily rescaled to adapt them to an adult woman or a child. However, the motor housing would need to be redesigned in order to fit the prosthesis with a socket for the patient’s arm stump. A possible solution to address this issue, in case of transradial amputation, was tested in a simulation environment and consists of a different version of the “Federica” hand that included the servomotor inside the palm (see Figure 15). The layout of both the palm and the mechanical components was changed; in particular, the palm was extended and raised, in order to house the servomotor.

**Figure 16:**
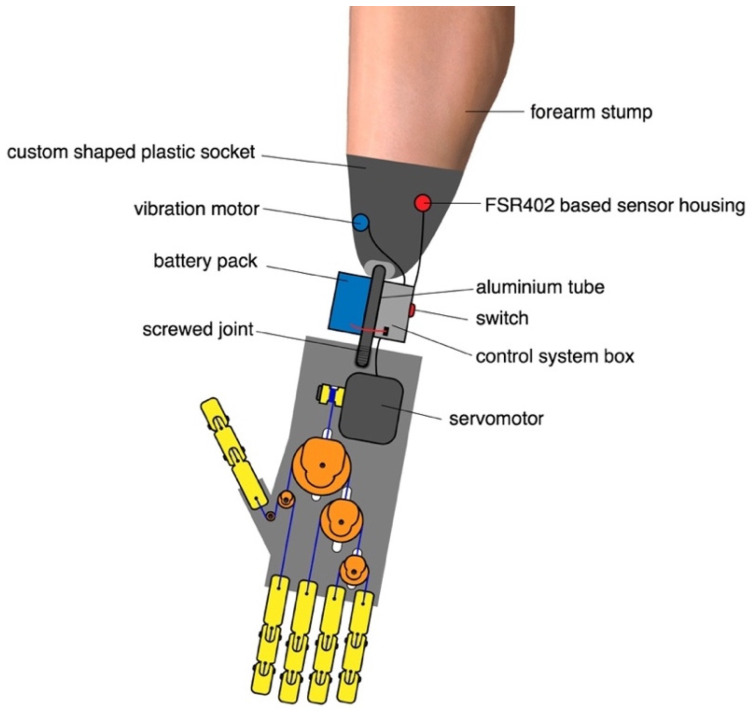
Illustration of a possible configuration of the prosthetic device on an amputee patient with transradial amputation. The arrangement consists of a 3D-printed socket and a version of “Federica” hand with built-in servomotor. The figure also shows the approximate positions of the various components (FSR-based sensor, vibration motor, control system, battery pack).

Figure 16 illustrates a possible arrangement of the prosthetic device combined with a 3D-printed socket for the arm stump. In the proposed design, the socket embeds the FSR-based sensor housing, in correspondence with the muscle assigned to the control. An aluminum tube, fastened in the distal part of the socket, ends with a screwed joint which is fixed in an ad-hoc slot of the hand prosthesis. The vibration motor for providing the sensory feedback of the grip force [9,30], is attached to the external surface of the socket. The battery pack (removable) and the control system box are hooked around the aluminum tube. Finally, the switch to activate the device is located on the control system box.

Each prosthesis socket should be custom-made for the specific patient, according to his/her level of amputation. Indeed, in the case of a long transradial amputation, it would be necessary to design alternative solutions for the positioning of the battery pack and the control system.

## 5. Conclusions

The “Federica” hand is a low-cost device, which presents various innovative aspects and advantageous solutions that can potentially be adopted by other projects.

The mechanical differential system provides a balanced force distribution to the fingers and allows a secure grip on a great variety of objects, regardless of their shape and deformability. The absence of return springs (used in many prosthetic devices for passive hand-opening [34,35]) and the closed-loop chain of tendons contribute to the high energy-transmission efficiency of the prosthesis.

Force-myography offers a valid alternative to EMG and is increasingly used for the control of human–machine interfaces, as showed in recent surveys [45,46]. Moreover, a further study [47] restates the high percentage of total rejection of EMG hand prostheses or the passive use of such devices, due to the control difficulties encountered by users. Therefore, it is important to seek and test new simpler solutions for prosthetic control.

The FSR-based sensor is extremely cheap, thin and can be easily placed on target muscles. FSR can operate in wet environments and does not suffer from electromagnetic interferences. The FSR provides a ready-to-use signal that does not require any processing, unlike EMG. By using FSR sensors, the training phase seems to be much simpler and faster for users. Furthermore, in spite of the electromechanical delay, FMG’s muscle-onset timing does not differ very much compared with that provided by EMG [14]. This contributes to the remarkable activation speed of the “Federica” hand.

The significant activation speed of the “Federica” hand is due to both its mechanical design and the FMG control system. Even considering the performances of recent and sophisticated prostheses available on market (0.35 s for Michelangelo by Ottobock, 0.80 s for iLimb Quantum by Touch Bionics, 1s for BesBionicV3 by Steeper) [34,35,48,49], the “Federica” hand takes about 0.5 s from the muscle-sensor trigger, until to the complete closure of the hand [11]. As further evidence, experimental tests carried out on healthy subjects showed the capability to grab objects on the fly [9] after a very short training.

“Federica” hand can provide the user with tactile sensory feedback of grip force, by means of a small vibrating motor, which can be also inserted in the prosthesis socket. Not all the prostheses provide feedback, and the user is enforced to use only visual feedback to ensure that the hold has occurred. Restoring tactile sensation in hand prostheses is helpful for improving user experience and manipulation performances [30].

Finally, the cost to acquire the “Federica” hand is considerably lower than commercial devices and also than many other prototypes [34,35]. Obviously, the cost of the manual labor for assembling parts was not estimated. However, the low cost and the relative simplicity of assembly make the “Federica” hand ideal for use in poor and developing countries.

Certainly, many improvements could still be added. First, an aesthetic silicone glove would be useful for better device acceptance. At present, the device allows only one grip modality, a power grasp with the simultaneous activation of all five fingers. However, “Federica”, even with its sole function of power grasping, is an adaptive hand prosthesis able to wrap objects and maintain a large contact area, and can be of help in many activities of daily living (ADL). A study [38] showed that power grasp actions achievable with “Federica”, such as medium wrap (i.e., grasping a cylinder) and power sphere (grasping a sphere), cover about 40% of the required grasps for ADL. A further study [50] showed for three ADLs, the percentage to which the power grasp is relevant for food preparation (30%), housekeeping (52%) and laundry (56%).

Obviously, “Federica” could be improved in functionality, by increasing the complexity of its mechanical and control system, to perform other grasps actions such the intermediate and precision ones. We believe that making the “Federica” hand an open project can offer some help to those in need and can stimulate other researchers or technicians to bring new ideas and solutions.

## Figures and Tables

**Table 1:** Design specifications for the “Federica” hand.

ITEM	SPECIFICATION
size	suitable for an adult man
mass	≈200 g (the only prosthetic hand)
modularity	yes
number of actuators	1
degrees of freedom	15
sensing of grip force	vibrotactile
max tested load	1 kg
activation speed (from trigger to complete closure)	≈0.5 s
energy power	2 × 3.7 V batteries (3000 mA h)
mean grip force	8.8 N
mean hysteresis for a complete cycle of closing–opening	106.80 Nm
force transfer ratio	12.85%

**Table 2:** List of components (related costs in USD) used for the realization of the “Federica” hand.

Component	Cost (USD)
3D-printed components (PLA)	20
other mechanical components (cables, screws…)	5
servomotor (Hitec HSR-5990TG) or equivalent	35
Arduino nano (processing unit board)	10
battery pack (2 × 3.7 V rechargeable battery)	10
piezoresistive force sensor (FSR 402 short)	5
electronic components (BJTs, resistors, capacitors, switch)	5
vibration motor	(5)
current sensor (ACS712-5A)	(5)
**Total**	**90 (100)**

**Table 3:** Comparison between “Federica” hand and other externally powered hand prostheses (prototypes and commercial devices [34]—highlighted in grey background).

	Developer/Manufacturer	Weight	Size (Height)	Grasp Speed	Grip Force	DOF	Kind/Number of Actuators	Power—Intermediate-Precision (Grasp)	Adaptive Grip	Price	Other Characteristics
Federica Hand	University of Naples “Federico II”, Italy	≈200 g	24 cm	≈0.5 s	≈9 N	15	1 servo	yes—no—no	yes	100 $	- tendon-based actuation - vibrotactile feedback
SmartHand [39]	ARTS Laboratory, Pontedera, Italy	520 g	-	≈2 s	≈36 N	16	4 brushed DC motors	yes—yes—yes	yes	-	tendon/spring based actuation
Remedi Hand [40]	University of Southampton, UK	400	human hand-sized	0.84 s	≈38 N	6	6 DC motors	yes—yes—yes	no	-	bar-linkage mechanism
UB Hand IV [41]	University of Bologna, Italy	-	human hand-sized	-	-	20	24 twisted-string actuators	yes—yes—yes	yes	-	tendon-based actuation
TBM Hand [42]	University of Toronto, CDN	280	14.6 cm	≈5 s	-	6	1 DC motor	yes—no—no	yes	-	compliant springs
MANUS hand [43]	Spain/Belgium/Israel	1200 g	-	-	-	3	2 brushless DC motors	yes—no—yes	no	-	crossed-tendons transmission
Vanderbilt Hand [44]	Vanderbilt University, Tennessee, US	580 g	19 cm	≈200 °/s	≈ 80 N	16	5 brushed DC servomotors	yes—no—yes	yes	-	tendon-based actuation
The SPRING hand [18]	ARTS Laboratory, Pontedera, Italy	-	-	-	<9 N	8	1 DC motor	yes—no—no	yes	-	- tendon-based actuation - 3 fingers prosthesis
TUAT/Karlsruhe Humanoid Hand [22]	College of Industrial Technology, Tokyo, Japan	-	17.5 cm	-	-	24	1 main servo + 6 sub-servos	yes—no—yes	yes	-	1 sub-servo for each finger and 2 for thumb
The KIT prosthetic hand [23]	Institute of Technology, Karlsruhe, Germany	-	-	1.3 s	≈24 N	10	2 DC motors	yes—no—no	yes	1000 €	video camera in the palm
ROBIOSS hand [25]	PPRIME, Poitiers University, France	-	-	70 °/s	-	16	16 DC motors	yes—no—yes	yes	-	- tendon-based actuation - 4 fingers prosthesis
Michelangelo	Ottobock	420 g	human hand-sized	0.35 s	70 N	2	2	yes—yes—no	no	60,000 $	cam design
Sensor Hand	Ottobock	500 g	-	300 mm/s	100 N	1	1 DC motor	yes—no—no	no	-	fixed pinch
Vincent Hand	Vincent Systems	-	-	-	-	6	6 DC motors-worm gear	yes—no—yes	yes	-	bar-linkage mechanism
iLimb Pulse	Touch Bionics	460 g	18.2 cm	1.2 s	136 N	6	5 DC motors-worm gear	yes—no—yes	yes	-	tendon-based actuation
Bebionic v2	RSL Steeper	540 g	20 cm	0.9 s	75	6	5 DC motors—lead screw	yes—no—yes	yes	-	bar-linkage mechanism

**Table 4:** Work for hand opening, closing and hysteresis [Nmm] of different body-powered hand/hook prostheses. The acronym VO stands for voluntarily open, while VC stands for voluntarily closed. The hook prostheses are highlighted with a grey background (Table extracted from [24]).

Prosthesis	Work for Hand Closing [Nmm] (Mean Value ± SD)	Work for Hand Opening [Nmm] (Mean Value ± SD)	Hysteresis [Nmm] (Mean Value ± SD)
“Federica” Hand	302.17 ± 4.42	196.84 ± 5.91	106.80 ± 3.31
Hosmer APRL VC Hand 52541	1058 ± 4	-	298 ± 8
Hosmer Soft VC hand 61794	2292 ± 12	-	1409 ± 37
Otto Bock VC 8K24, frame	1624 ± 8	-	389 ± 19
Hosmer Sierra VO Hand (ungloved)	-	1152 ± 8	637 ± 6
RSL Steeper VO Hand (ungloved)	-	1758 ± 27	855 ± 6
Otto Bock VO Hand (ungloved)	-	2545 ± 11	917 ± 5
Hosmer Becker VO (ungloved)	-	2748 ± 17	1710 ± 9
RSL Steeper Carbon VO	-	1619 ± 2	487 ± 4
Hosmer APRL VC Hook 52601	720 ± 6	-	138 ± 3
TSR VC Hook-Grip 2SS	284 ± 3	-	52 ± 1
Hosmer Model VO 5XA Hook (1 band)	-	1128 ± 14	290 ± 3
Otto Bock VO 10A60 Hook	-	1002 ± 3	482 ± 5
Hosmer Sierra 2 Load VO Hook	-	1243 ± 11	379 ± 1

## Data Availability

Further information about the “Federica” hand (e.g., videos) can be found at http://ingegneria-biomedica.dieti.unina.it/index.php/en/projects/federica-prosthetic-hand.html (accessed on 16 September 2021).

## Supplementary Materials

All the information to reproduce the “Federica” hand (including CAD files and software) can be downloaded from: https://www.doi.org/10.6084/m9.figshare.13480248 (accessed on 16 September 2021).
